# Vagus Nerve Stimulation Attenuates Hepatic Ischemia/Reperfusion Injury via the Nrf2/HO-1 Pathway

**DOI:** 10.1155/2019/9549506

**Published:** 2019-05-07

**Authors:** Qianqian Zhang, Yanqiu Lai, Jielin Deng, Menglong Wang, Zhenya Wang, Meng Wang, Yifeng Zhang, Xiaomeng Yang, Xiaoya Zhou, Hong Jiang

**Affiliations:** Department of Cardiology, Renmin Hospital of Wuhan University, Cardiovascular Research Institute, Wuhan University, Hubei Key Laboratory of Cardiology, Wuhan 430060, China

## Abstract

It has been demonstrated that vagus nerve stimulation (VNS) plays a protective role in ischemia/reperfusion (I/R) injury of various organs. The present study investigates the protective effect of VNS on hepatic I/R injury and the potential mechanisms. Male Sprague-Dawley rats were randomly allocated into three groups: the sham operation group (Sham; *n* = 6, sham surgery with sham VNS); the I/R group (*n* = 6, hepatic I/R surgery with sham VNS); and the VNS group (*n* = 6, hepatic I/R surgery plus VNS). The I/R model was established by 1 hour of 70% hepatic ischemia. Tissue samples and blood samples were collected after 6 hours of reperfusion. The left cervical vagus nerve was separated and stimulated throughout the whole I/R process. The stimulus intensity was standardized to the voltage level that slowed the sinus rate by 10%. VNS significantly reduced the necrotic area and cell death in I/R tissues. Serum levels of alanine aminotransferase (ALT), aspartate aminotransferase (AST), and lactate dehydrogenase (LDH) were also decreased by VNS. In addition, VNS suppressed inflammation, oxidative stress, and apoptosis in I/R tissues. VNS significantly increased the protein levels of nuclear factor erythroid 2-related factor 2 (Nrf2)/heme oxygenase-1 (HO-1) in the liver. These data indicated that VNS may attenuate hepatic I/R injury by inhibiting inflammation, oxidative stress, and apoptosis possibly via the Nrf2/HO-1 pathway.

## 1. Introduction

Hepatic ischemia/reperfusion (I/R) damage often occurs during liver surgery procedures, such as liver resection and liver transplantation [1]. Despite the rapid technological advances in liver surgery, hepatic I/R injury remains a critical concern and can lead to liver dysfunction, remote organ failure, and high morbidity and mortality rates [2]. Unfortunately, the curative effect of the current therapeutic strategies is still very limited. Therefore, seeking novel effective treatments to prevent this injury is necessary.

As a transcription factor, nuclear factor erythroid 2-related factor 2 (nrf2) participates in regulating oxidative stress in organs. Under physiological conditions, Nrf2 is mainly localized in the cytoplasm. When activated by various stimuli, Nrf2 transfers into the nucleus and upregulates relevant cytoprotective enzymes, including heme oxygenase-1 (HO-1) [3, 4]. Many researches indicate that the Nrf2/HO-1 pathway is closely involved in alleviating hepatic I/R injury [5–7].

A number of studies have demonstrated that vagus nerve stimulation (VNS) plays a protective role in I/R injury of the kidney, heart, and other organs [8–10]. Hepatic vagotomy has been reported to aggravate hepatic injury and cell apoptosis induced by I/R, indicating the protective role of the hepatic vagus nerve [11]. Furthermore, *α*7nAChR activation has been shown to enhance the Nrf2/HO-1 pathway [12–15]. Unfortunately, it is still unclear whether VNS can protect against hepatic I/R injury. In the current experiment, an acute model of hepatic I/R injury in rats was used to evaluate the protective effect of VNS, and the potential mechanisms were explored.

## 2. Materials and Methods

### 2.1. Animal Preparation

All experimental operations were approved by the Animal Care and Use Committee of Wuhan University and strictly conformed to the Guide for the Care and Use of Laboratory Animals. The male Sprague-Dawley rats (each weighing 220-260 g) used in the present study were purchased from the Animal Center of Wuhan University. Eighteen rats were allocated into three groups: the sham operation group (Sham; *n* = 6); the I/R group (*n* = 6); and the I/R+VNS (VNS) group (*n* = 6). The rats were housed in cages with an alternating 12-hour light/dark cycle and a controlled temperature (24°C) and had no restriction on food and water. All animals were fasted for 8 hours before surgeries. During the experiment, a surface electrocardiogram in rats was recorded with a BIOPAC system (MP150, Goleta, USA).

### 2.2. Model of Hepatic I/R Injury

**Figure 1:**
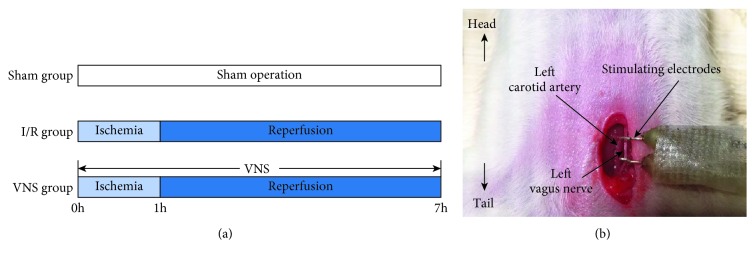
Schematic illustration of the (a) experimental protocol and (b) location of the left vagus nerve. Sham: sham operation; I/R: ischemia-reperfusion; VNS: vagus nerve stimulation.

An acute model of segmental (70%) hepatic ischemia was established according to Ni et al. [11]. All rats were anesthetized with pentobarbital (1%, 40 mg/kg) intraperitoneally. After a midline laparotomy was performed, the portal triad to the left and median liver lobes was separated and occluded by a noninvasive vascular clamp. The partial hepatic ischemia lasted for 1 hour. The clamp was then removed, and the abdominal wound was sewed. After 6 hours of reperfusion, blood samples from the portal vein and liver tissues from the ischemic lobes were collected for further detections. Animals in the Sham group underwent the same surgery except the occlusion. The protocol is outlined in Figure 1(a).

### 2.3. Vagus Nerve Stimulation

The hepatic vagus branches originate from the left vagus nerve. Therefore, we chose the left vagus nerve as the stimulating target. Left cervical incision was performed to expose the left cervical vagal trunk. A pair of self-made silver electrodes and a stimulator (S20, Jinjiang, Chengdu City, China) were used to deliver the high-frequency stimulation (HFS) (see Figure 1(b)). The stimulus frequency was 20 Hz, and the duration was 0.1 millisecond. The stimulus intensity was standardized to the voltage level that slowed the sinus rate by 10% and adjusted each hour, according to Liu et al. [16]. In the Sham group and the I/R group, the vagus nerve was exposed and the electrodes were placed, while no electrical stimulation was delivered.

### 2.4. Histological Examinations

Liver damage was detected by histological examinations. The liver tissues of the ischemic lobes were fixed with paraformaldehyde and embedded in paraffin. The tissues were cut into sections (4 *μ*m thick) with a microtome and stained with hematoxylin and eosin (H&E). Suzuki classification was used to grade histological injury from 0 to 4, according to the degree of sinusoidal congestion, vacuolization of hepatocyte cytoplasm, and hepatocyte necrosis [17]. The percentage of the necrotic area was quantified in at least 5 fields per liver sample. Terminal deoxynucleotidyl transferase dUTP nick end labeling (TUNEL) staining was performed to determine hepatocyte apoptosis, according to the manufacturer's protocol (Roche, Shanghai, China). Liver sections were dewaxed in xylene and then hydrated with graded ethanol. After being treated with protease K (20 *μ*g/mL), the sections were washed in phosphate-buffered saline (PBS). The liver sections were then incubated in a TUNEL reagent for 60 minutes at 37°C in the dark and washed again. After that, 4′,6-diamino-2-phenylindole (DAPI) was used and the sections were incubated in the dark. The percentage of TUNEL-positive hepatocytes was calculated in 3 random high-power fields under an inverted fluorescence microscope (Nikon ECLIPSE TI-SR, Japan).

### 2.5. Serum Biochemical Measurements

Blood samples were collected and centrifuged to separate the serum (3,000 rpm, 15 minutes). Levels of alanine aminotransferase (ALT), aspartate aminotransferase (AST), and lactate dehydrogenase (LDH) were detected using the ADVIA 2400 Chemistry System (Siemens, Erlangen, Germany).

### 2.6. Liver Biochemical Measurements

Levels of malondialdehyde (MDA) and glutathione (GSH) and activities of superoxide dismutase (SOD) and catalase (CAT) were measured in the liver homogenate using different assay kits (Nanjing Jiancheng Bioengineering Institute). The concentrations of GSH and MDA were estimated according to the instructions of the respective kits and were reported as nmol/mg protein. SOD activity was tested by the xanthine oxidase technique, and CAT activity was detected by a peroxide indicator assay; both were expressed as U/mg protein.

### 2.7. Quantitative Real-Time Polymerase Chain Reaction (RT-PCR)

Total mRNA was extracted from liver tissues using a TRIpure reagent (ELK Biotechnology) according to the manufacturer's protocols. The synthesis of first-strand cDNA was performed using M-MLV Reverse Transcriptase (ELK Biotechnology). The expression levels of the target genes were measured with EnTurbo™ SYBR Green PCR SuperMix (ELK Biotechnology) using a StepOne™ RT-PCR thermocycler (Life Technologies). The mRNA level of each gene was normalized to the *β*-actin mRNA level of the same sample using the delta-delta CT method. All RT-PCR primer sequences are listed as follows: IL-1*β* 5′-ATGAAAGACGGCACACCCAC-3′ and 5′-GGTGCTGATGTACCAGTTGGG-3′; IL-6 5′-GCCAGAGTCATTCAGAGCAAT-3′ and 5′-CTTGGTCCTTAGCCACTCCT-3′; TNF-*α* 5′-CACCACGCTCTTCTGTCTACTG-3′ and 5′-GCTACGGGCTTGTCACTCG-3′; and *β*-actin 5′-CGTTGACATCCGTAAAGACCTC-3′ and 5′-TAGGAGCCAGGGCAGTAATCT-3′.

### 2.8. Western Blotting Analysis

The levels of Bcl-2, Bax, nuclear factor (NF)-*κ*B p65, phosphorylated p65 (p-p65), and HO-1 were measured in liver lysates, and the level of Nrf2 was detected in nuclear lysates. A protein sample from each rat was determined. And the protein expression of each sample was visualized as a protein band. Briefly, equal amounts of denatured protein were separated by SDS-PAGE. Then, the protein was transferred to a polyvinylidene fluoride (PVDF) membrane. After that, the membranes were blocked with 5% nonfat milk for 1 hour and incubated with primary antibodies (Bcl-2, Abcam; Bax, CST; HO-1, Abcam; NF-*κ*B p65, Abcam; p-p65, Abcam; Nrf2, Abcam; *β*-actin, Abcam; and Histone H3, CST) at 4°C overnight. Membranes were then washed in TBST and incubated with anti-rabbit secondary antibodies. The relative protein expression was normalized to *β*-actin (total protein) or histone H3 (nucleoprotein) and quantified with image analyzer software (AlphaEase FC, USA).

### 2.9. Statistical Analysis

All continuous data are expressed as the mean ± SD. Comparisons between the different groups were performed by one-way ANOVA. The data were analyzed with GraphPad Prism 6.0 software. Differences were considered to be significant at *p* < 0.05.

## 3. Results

### 3.1. VNS Ameliorated Hepatic I/R Injury

**Figure 2:**
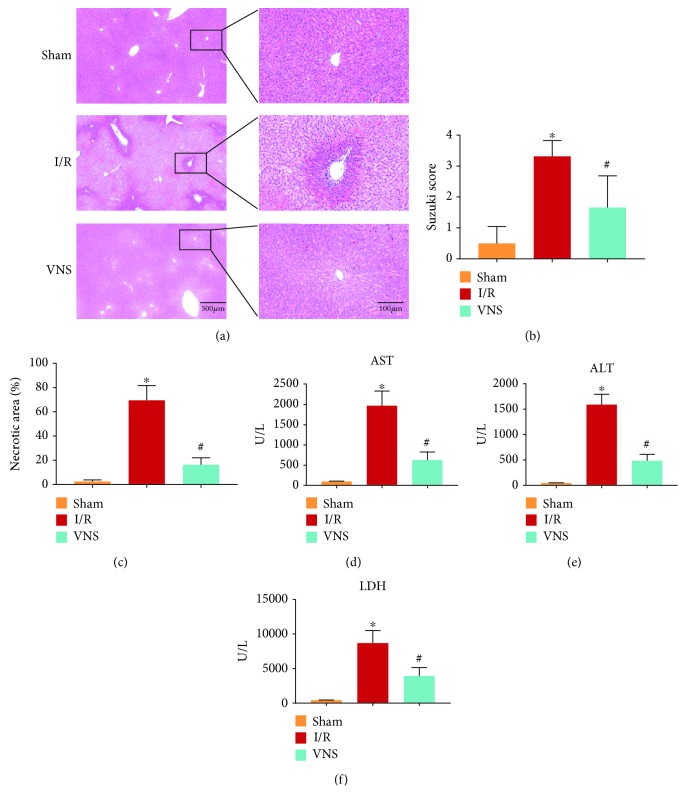
VNS ameliorated hepatic I/R injury. (a) Representative H&E staining images of liver tissues show necrotic areas in the three groups. (b) The Suzuki scores and (c) the percentages of necrotic liver sections are shown. Levels of serum (d) AST, (e) ALT, and (f) LDH are presented. ^∗^*p* < 0.05 vs. the Sham group; ^#^*p* < 0.05 vs. the I/R group. AST: aspartate aminotransferase; ALT: alanine aminotransferase; LDH: lactate dehydrogenase.

**Figure 3:**
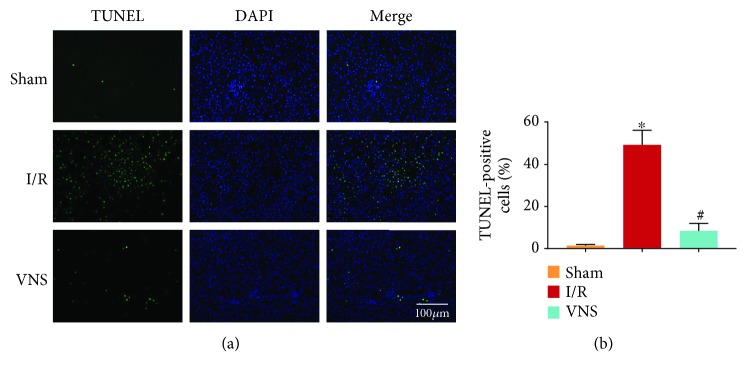
VNS induced hepatocyte apoptosis. (a) Representative micrographs of immunofluorescence staining for TUNEL (green) and DAPI (blue) in hepatocyte nuclei from the three groups. (b) The percentages of TUNEL-positive hepatocytes are shown. ^∗^*p* < 0.05 vs. the Sham group; ^#^*p* < 0.05 vs. the I/R group.

Histological examinations and blood detections were performed to determine hepatocellular damage. The necrotic area was dramatically enlarged in the I/R group, as shown by H&E staining (see Figures 2(a)–2(c)). The VNS group showed a marked reduction in necrotic liver tissue compared to the I/R group (see Figures 2(a)–2(c)). In parallel, higher Suzuki scores were observed in the I/R group compared to those in the Sham group (see Figure 2(b)). Additionally, Suzuki scores were lowered by VNS (see Figure 2(b)). Consistent with the histological alterations, hepatic I/R markedly elevated serum AST, ALT, and LDH levels (see Figures 2(d) and 2(e)), indicating impaired liver function. The VNS group exhibited a reversal of this dysfunction, which was evidenced by lower levels of AST, ALT, and LDH (see Figures 2(d) and 2(e)). TUNEL-positive hepatocytes were increased in the I/R group (see Figures 3(a) and 3(b)). On the contrary, the percentage of TUNEL-positive hepatocytes was markedly reduced in the VNS group compared to that in the I/R group (see Figures 3(a) and 3(b)). These findings indicated that VNS improved hepatic I/R injury.

### 3.2. VNS Reduced Hepatic Apoptosis by Regulating the Expression of Bcl-2 and Bax

**Figure 4:**
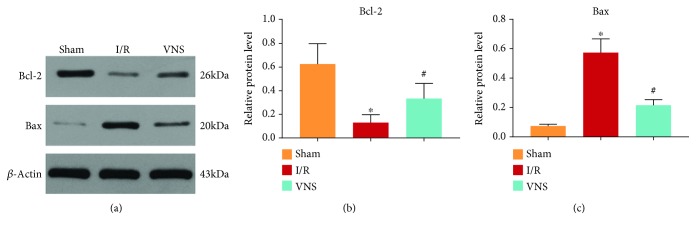
VNS reduced hepatic apoptosis by regulating Bcl-2 and Bax. (a) Representative blots and relative protein levels of (b) Bcl-2 and (c) Bax in liver tissues are shown. ^∗^*p* < 0.05 vs. the Sham group; ^#^*p* < 0.05 vs. the I/R group.

To evaluate the cell apoptosis in the ischemic tissues, the expression levels of Bcl-2 and Bax were detected. In the I/R group, the level of Bcl-2, which inhibits apoptosis, was significantly decreased (see Figures 4(a) and 4(b)). Moreover, the level of Bax, which induces apoptosis, was increased (see Figures 4(a) and 4(c)). In contrast, VNS reversed these changes (see Figures 4(a)–4(c)). The above results indicated that VNS reduced the extent of apoptosis through Bcl-2 and Bax.

### 3.3. VNS Inhibited Inflammation in the Liver

**Figure 5:**
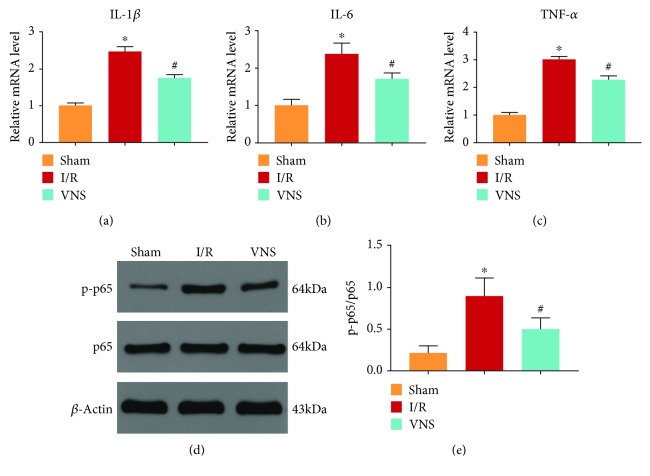
VNS inhibited inflammation in the liver. Relative mRNA levels of (a) IL-1*β*, (b) IL-6, and (c) TNF-*α* are shown. (d) Representative blots and (e) relative protein levels of NF-*κ*B in liver tissues from the different groups are shown. ^∗^*p* < 0.05 vs. the Sham group; ^#^*p* < 0.05 vs. the I/R group. IL-1*β*: interleukin-1*β*; IL-6: interleukin-6; TNF-*α*: tumor necrosis factor-*α*; p-p65: phosphorylated p65.

The mRNA levels of inflammatory cytokines were measured to evaluate inflammation in the liver. IL-1*β*, IL-6, and TNF-*α* mRNA levels were elevated in the I/R group (see Figures 5(a)–5(c)). On the contrary, VNS lowered the mRNA levels of those inflammatory cytokines (see Figures 5(a)–5(c)), compared with those in the I/R group. Additionally, hepatic I/R notably increased the protein expression of NF-*κ*B p-p65, and VNS reversed this increase (see Figures 5(d) and 5(e)). These results suggested that VNS inhibited inflammation induced by I/R injury.

### 3.4. VNS Alleviated Oxidative Stress in the Liver

**Figure 6:**
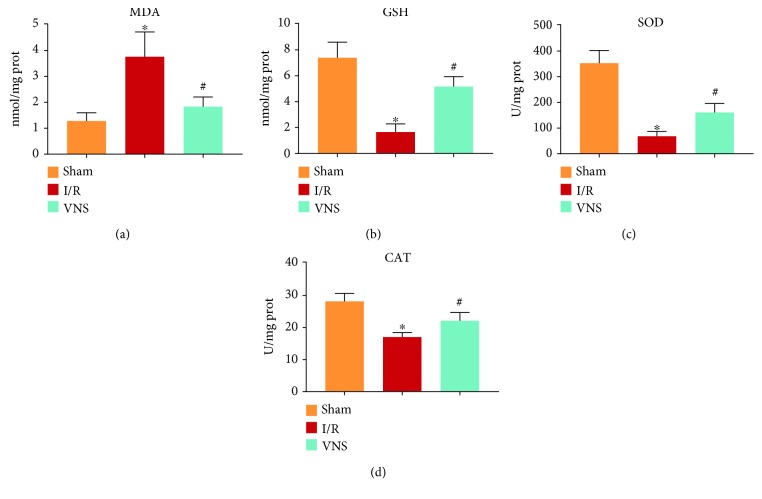
VNS alleviated oxidative stress in the liver. The effect of VNS on the levels of (a) MDA and (b) GSH in liver tissues is shown. The effect of VNS on the activity of (c) SOD and (d) CAT in liver tissues is shown. ^∗^*p* < 0.05 vs. the Sham group; ^#^*p* < 0.05 vs. the I/R group. MDA: malondialdehyde; GSH: glutathione; SOD: superoxide dismutase; CAT: catalase.

To evaluate the effect of VNS on hepatic oxidative stress, the levels of MDA and GSH, as well as SOD and CAT activities, were examined in the different groups. As a prooxidative stress indicator, MDA was increased significantly in the I/R group (see Figure 6(a)). However, this increase was reversed by VNS (see Figure 6(a)). The level of GSH, a powerful antioxidant, was noticeably reduced in the I/R group (see Figure 6(b)). In contrast, VNS elevated the level of GSH compared with that of I/R (see Figure 6(b)). The activities of SOD and CAT, two important antioxidant enzymes, were significantly inhibited in the I/R group (see Figures 6(c) and 6(d)). In contrast, the activities of these enzymes were enhanced in the VNS group compared with those in the I/R group (see Figures 6(c) and 6(d)). These results suggested that VNS reduced oxidative stress in the I/R liver.

### 3.5. VNS Activated the Nrf2/HO-1 Pathway in the Liver

**Figure 7:**
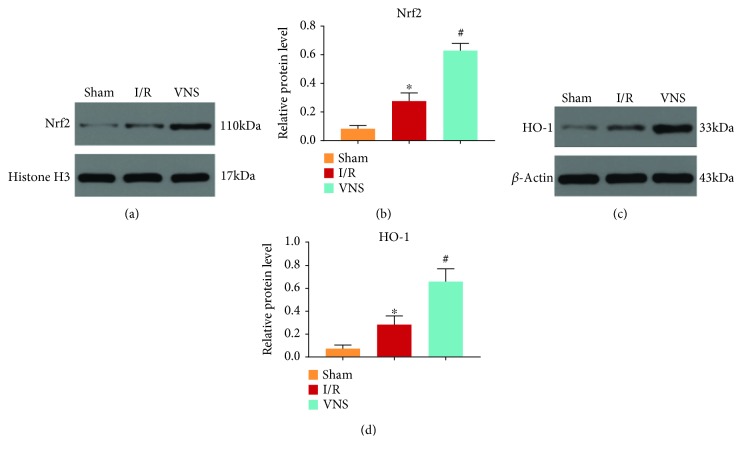
VNS activated the Nrf2/HO-1 pathway in the liver. (a) Representative blots and (b) relative protein levels of Nrf2 in liver tissues are shown. (c) Representative blots and (d) relative protein levels of HO-1 in liver tissues are shown. ^∗^*p* < 0.05 vs. the Sham group; ^#^*p* < 0.05 vs. the I/R group. Nrf2: nuclear factor erythroid 2-related factor 2; HO-1: heme oxygenase-1.

Hepatic I/R markedly increased the protein levels of Nrf2 and HO-1 (see Figures 7(a)–7(d)). And VNS markedly augmented these increases in the liver (see Figures 7(a)–7(d)). These results indicated that the activation of the Nrf2/HO-1 pathway induced in the I/R liver was enhanced by VNS.

## 4. Discussion

### 4.1. Major Findings

In the current study, VNS throughout the hepatic I/R procedure significantly attenuated liver injury. Serum AST, ALT, and LDH levels were decreased, and hepatocyte necrosis was reduced by VNS. Additionally, VNS inhibited inflammation, oxidative stress, and apoptosis induced by hepatic I/R. Furthermore, VNS markedly enhanced Nrf2/HO-1 signaling in the liver, which may be a potential mechanism underlying its effect.

### 4.2. VNS Protects against Hepatic I/R Injury

VNS is an FDA-approved clinical treatment for drug-resistant depression and medically intractable partial-onset seizures [18]. Accumulating evidence from animal experiments has indicated that VNS protects against I/R injury of various organs. Our previous study verified that VNS applied to dogs significantly reduced myocardial I/R injury [8]. Another study [9] also reported that cerebral I/R injury could be alleviated by VNS in a rat model. Similarly, Inoue et al. [10] found that pretreatment of VNS markedly reduced acute kidney injury in mice. Considering the similarity of physiopathological processes in different I/R organs, we hypothesize that VNS can also play a protective role in hepatic I/R. Furthermore, many studies have revealed the importance of the autonomic nervous system in several liver injuries, including I/R injury. It has been proven that the sympathetic nervous system participates in raising oxidative stress in the liver. Lin et al. [19] have reported that hepatic oxidative stress induced by carbon tetrachloride injection was relieved in sympathectomized mice. Oben et al. [20] have also reported that 6-hydroxydopamine, an agent used to induce chemical sympathectomy, significantly improved liver injury in mice with antioxidant-depleted diets. The vagus nerve system, as a natural antagonist of the sympathetic nervous system, has been demonstrated to play a vital role in ameliorating hepatic I/R injury. Ni et al. [11] found that both hepatic vagotomy and *α*7nAChR^−/−^ could aggravate I/R-induced liver apoptosis in mice. Moreover, numerous researches have suggested that pretreatment with different acetylcholine receptor agonists significantly reduced liver I/R injury in mice [15, 21, 22]. These results suggest that the vagus nerve system may protect the liver by activating *α*7nAChR. The present study first reported that direct VNS can effectively protect against hepatic I/R injury, consistent with these previous studies.

### 4.3. Potential Mechanisms

Among a considerable variety of underlying mechanisms in hepatic I/R injury, increased inflammatory reactions play a pivotal part [5, 23]. The VNS-mediated cholinergic anti-inflammatory pathway has been well studied in the liver. Acetylcholine released by vagus nerve terminals can bind to *α*7nAChR on Kupffer cells and prevent the production of inflammatory cytokines [24, 25]. Several studies have reported that treatment with *α*7nAChR agonists significantly inhibited hepatic NF-*κ*B activation in the I/R liver [15, 21]. In the current study, the inflammation factor levels and NF-*κ*B signaling in I/R tissues were reduced by VNS, indicating that the cholinergic anti-inflammatory pathway may be a potential mechanism.

Excessive production of reactive oxygen species in the early phase of reperfusion is another critical factor in hepatic I/R injury [26, 27]. Some prior researches have indicated that the activation of antioxidative enzymes and antioxidants significantly ameliorated hepatic I/R injury [28, 29]. Furthermore, our previous study showed that VNS reduced the level of MDA and increased the activity of SOD in a canine myocardial I/R model [8]. The present study indicated that VNS decreased the level of MDA, elevated the level of GSH, and activated SOD and CAT in the I/R liver. These data suggest that VNS protects the I/R liver partly through its antioxidative properties.

The Bcl-2 family, a crucial regulator of apoptosis, plays a key role in I/R. Bcl-2 (an antiapoptotic protein) and Bax (a proapoptotic protein) are well known to participate in the occurrence of cell death in the I/R liver [30]. Our previous study verified the regulatory effect of VNS on the Bcl-2 family. VNS applied to myocardial I/R dogs markedly induced the expression of Bcl-2 while inhibiting the expression of Bax [8]. Similarly, in the present study, VNS exerted an antiapoptotic effect through the Bcl-2 family. These results indicate that the Bcl-2 family may be involved in the mechanisms of the protection of VNS.

To investigate the deeper molecular mechanisms, the expression levels of Nrf2 and HO-1 in I/R tissues were measured. Accumulating evidence from studies has indicated the protective effect of the Nrf2/HO-1 pathway against hepatic I/R damage [5–7]. Kudoh et al. reported that depletion of Nrf2 in mice aggravated inflammation, oxidative stress, and cell apoptosis in the I/R liver, while activation of Nrf2 significantly suppressed hepatic damage [7]. Moreover, a large number of researches have demonstrated that the Nrf2/HO-1 pathway can be induced by *α*7nAChR activation [12–15]. Navarro et al. found that Nrf2 deletion removed the neuroprotective effect of PNU282987, an *α*7nAChR agonist [14]. Furthermore, in a hepatic I/R model, nicotine treatment activated the Nrf2/HO-1 pathway and reduced I/R injury. However, zinc protoporphyrin, an HO-1 inhibitor, eliminated this protection [15]. These results indicate that *α*7nAChR activation exerts its protective effect in an Nrf2-dependent manner. Consistent with previous reports, our study showed that VNS could significantly increase Nrf2/HO-1 signaling in the I/R liver. Therefore, the Nrf2/HO-1 pathway may act as a pivotal mediator for the protective effects of VNS on hepatic I/R.

### 4.4. Clinical Implications

Hepatic I/R injury often occurs in liver surgeries and remains a lethal threat. Here, we demonstrated the protective effect of VNS on hepatic I/R damage. VNS has been widely used for the treatment of drug-resistant epilepsy and depression. Traditional VNS requires device implantation, which limited its application. Recent studies have demonstrated that auricular VNS, a noninvasive stimulation of the vagus nerve, could achieve the same effects as VNS [31, 32]. Therefore, this type of noninvasive VNS might be a novel therapeutic measure for hepatic I/R in patients.

### 4.5. Study Limitations

There are several limitations in this study. First, we used pentobarbital for anesthesia, which may influence the autonomic nerve system. However, the experiments in the three groups were performed under the same anesthetic condition to eliminate this influence. Second, the present study only revealed the activation of the Nrf2/HO-1 pathway after VNS. The effect of VNS after inhibition of Nrf2/HO-1 is supposed to be explored in our further studies. Third, we delivered VNS only with the current parameters. Further studies are needed to investigate the most appropriate stimulation parameters. Last, we only explored the acute effect of VNS on hepatic I/R injury in the present study. The long-term impact remains unclear.

## 5. Conclusions

**Figure 8:**
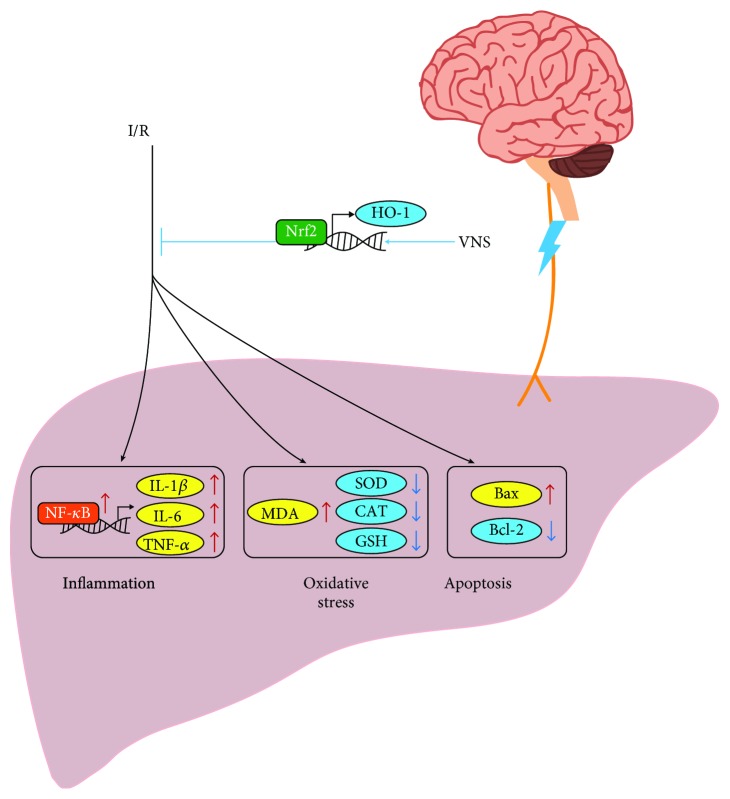
Schematic illustration of the protective effect of VNS on hepatic I/R injury and potential mechanisms. VNS protects against hepatic I/R injury by inhibiting inflammation, oxidative stress, and apoptosis in the liver, possibly via the Nrf2/HO-1 pathway.

In conclusion, we provided evidence that VNS applied to an acute hepatic I/R model significantly attenuated hepatic I/R injury by inhibiting inflammation, oxidative stress, and apoptosis in the liver. In addition, we found that VNS markedly activated the Nrf2/HO-1 pathway in the liver. Considering these results together, the present experiment demonstrated that VNS may protect against hepatic I/R injury by enhancing Nrf2/HO-1 signaling (see Figure 8).

## Data Availability

The data used to support the findings of this study are available from the corresponding authors upon request.

## References

[1] Zhai Y., Petrowsky H., Hong J. C., Busuttil R. W., Kupiec-Weglinski J. W. (2013). Ischaemia–reperfusion injury in liver transplantation—from bench to bedside. *Nature Reviews Gastroenterology & Hepatology*.

[2] Lentsch A. B., Kato A., Yoshidome H., McMasters K. M., Edwards M. J. (2000). Inflammatory mechanisms and therapeutic strategies for warm hepatic ischemia/reperfusion injury. *Hepatology*.

[3] Rojo de la Vega M., Chapman E., Zhang D. D. (2018). NRF2 and the hallmarks of cancer. *Cancer Cell*.

[4] Kensler T. W., Wakabayashi N., Biswal S. (2007). Cell survival responses to environmental stresses via the Keap1-Nrf2-ARE pathway. *Annual Review of Pharmacology and Toxicology*.

[5] Nakamura K., Zhang M., Kageyama S. (2017). Macrophage heme oxygenase-1-SIRT1-p53 axis regulates sterile inflammation in liver ischemia-reperfusion injury. *Journal of Hepatology*.

[6] Ke B., Shen X. D., Zhang Y. (2013). KEAP1-NRF2 complex in ischemia-induced hepatocellular damage of mouse liver transplants. *Journal of Hepatology*.

[7] Kudoh K., Uchinami H., Yoshioka M., Seki E., Yamamoto Y. (2014). Nrf2 activation protects the liver from ischemia/reperfusion injury in mice. *Annals of Surgery*.

[8] Chen M., Zhou X., Yu L. (2016). Low-level vagus nerve stimulation attenuates myocardial ischemic reperfusion injury by antioxidative stress and antiapoptosis reactions in canines. *Journal of Cardiovascular Electrophysiology*.

[9] Jiang Y., Li L., Liu B., Zhang Y., Chen Q., Li C. (2015). PPAR*γ* upregulation induced by vagus nerve stimulation exerts anti-inflammatory effect in cerebral ischemia/reperfusion rats. *Medical Science Monitor*.

[10] Inoue T., Abe C., Sung S.-s. J. (2016). Vagus nerve stimulation mediates protection from kidney ischemia-reperfusion injury through *α*7nAChR^+^ splenocytes. *The Journal of Clinical Investigation*.

[11] Ni M., Fu H., Huang F. (2016). Vagus nerve attenuates hepatocyte apoptosis upon ischemia–reperfusion via *α*7 nicotinic acetylcholine receptor on Kupffer cells in mice. *Anesthesiology*.

[12] Egea J., Buendia I., Parada E., Navarro E., Leon R., Lopez M. G. (2015). Anti-inflammatory role of microglial alpha7 nAChRs and its role in neuroprotection. *Biochemical Pharmacology*.

[13] Kalkman H. O., Feuerbach D. (2016). Modulatory effects of *α*7 nAChRs on the immune system and its relevance for CNS disorders. *Cellular and Molecular Life Sciences*.

[14] Navarro E., Gonzalez-Lafuente L., Perez-Liebana I. (2017). Heme-oxygenase I and PCG-1*α* regulate mitochondrial biogenesis via microglial activation of alpha7 nicotinic acetylcholine receptors using PNU282987. *Antioxidants & Redox Signaling*.

[15] Park J., Kang J. W., Lee S. M. (2013). Activation of the cholinergic anti-inflammatory pathway by nicotine attenuates hepatic ischemia/reperfusion injury via heme oxygenase-1 induction. *European Journal of Pharmacology*.

[16] Liu A. F., Zhao F. B., Wang J. (2016). Effects of vagus nerve stimulation on cognitive functioning in rats with cerebral ischemia reperfusion. *Journal of Translational Medicine*.

[17] Suzuki S., Toledo-Pereyra L. H., Rodriguez F. J., Cejalvo D. (1993). Neutrophil infiltration as an important factor in liver ischemia and reperfusion injury. Modulating effects of FK506 and cyclosporine. *Transplantation*.

[18] Shuchman M. (2007). Approving the vagus-nerve stimulator for depression. *The New England Journal of Medicine*.

[19] Lin J. C., Peng Y. J., Wang S. Y. (2016). Sympathetic nervous system control of carbon tetrachloride-induced oxidative stress in liver through *α*-adrenergic signaling. *Oxidative Medicine and Cellular Longevity*.

[20] Oben J. A., Roskams T., Yang S. (2003). Sympathetic nervous system inhibition increases hepatic progenitors and reduces liver injury. *Hepatology*.

[21] Li F., Chen Z., Pan Q. (2013). The protective effect of PNU-282987, a selective *α*7 nicotinic acetylcholine receptor agonist, on the hepatic ischemia-reperfusion injury is associated with the inhibition of high-mobility group box 1 protein expression and NF-*κ*B activation in mice. *Shock*.

[22] Crockett E. T., Galligan J. J., Uhal B. D., Harkema J., Roth R., Pandya K. (2006). Protection of early phase hepatic ischemia-reperfusion injury by cholinergic agonists. *BMC Clinical Pathology*.

[23] Nakao T., Ono Y., Dai H. (2018). DNAX activating protein of 12 kDa/triggering receptor expressed on myeloid cells 2 expression by mouse and human liver dendritic cells: functional implications and regulation of liver ischemia–reperfusion injury. *Hepatology*.

[24] Borovikova L. V., Ivanova S., Zhang M. (2000). Vagus nerve stimulation attenuates the systemic inflammatory response to endotoxin. *Nature*.

[25] Wang H., Yu M., Ochani M. (2003). Nicotinic acetylcholine receptor *α*7 subunit is an essential regulator of inflammation. *Nature*.

[26] Motino O., Frances D. E., Casanova N. (2018). Protective role of hepatocyte cyclooxygenase-2 expression against liver ischemia-reperfusion injury in mice. *Hepatology*.

[27] Chen H. H., Chen Y. T., Yang C. C. (2016). Melatonin pretreatment enhances the therapeutic effects of exogenous mitochondria against hepatic ischemia-reperfusion injury in rats through suppression of mitochondrial permeability transition. *Journal of Pineal Research*.

[28] He S. Q., Zhang Y. H., Venugopal S. K. (2006). Delivery of antioxidative enzyme genes protects against ischemia/reperfusion–induced liver injury in mice. *Liver Transplantation*.

[29] Schauer R. J., Gerbes A. L., Vonier D. (2004). Glutathione protects the rat liver against reperfusion injury after prolonged warm ischemia. *Annals of Surgery*.

[30] Jin S., Dai C. L. (2012). Attenuation of reperfusion-induced hepatocyte apoptosis is associated with reversed bcl-2/bax ratio in hemi-hepatic artery-preserved portal occlusion. *Journal of Surgical Research*.

[31] Yu L., Huang B., Po S. S. (2017). Low-level tragus stimulation for the treatment of ischemia and reperfusion injury in patients with ST-segment elevation myocardial infarction: a proof-of-concept study. *JACC: Cardiovascular Interventions*.

[32] Fang J., Rong P., Hong Y. (2016). Transcutaneous vagus nerve stimulation modulates default mode network in major depressive disorder. *Biological Psychiatry*.

